# Transtorno do espectro autista na atualidade: reflexões críticas sobre o hiperdiagnóstico

**DOI:** 10.1590/0102-311XPT140725

**Published:** 2026-05-18

**Authors:** Raphael Alves da Silva, Shelismania do Prado Oliveira Queiroz, Felicialle Pereira da Silva

**Affiliations:** 1 Hospital Otavio de Freitas, Recife, Brasil.; 2 Programa de Pós-graduação em Enfermagem em Pneumologia, Instituto de Medicina Integral Professor Fernando Figueira, Recife, Brasil.; 3 Faculdade de Enfermagem Nossa Senhora das Graças, Universidade de Pernambuco, Recife, Brasil.

**Keywords:** Transtorno do Espectro Autista, Diagnóstico, Sobrediagnóstico, Autism Spectrum Disorder, Diagnosis, Overdiagnosis, Trastorno del Espectro Autista, Diagnóstico, Sobrediagnóstico

## Abstract

Este artigo aborda o fenômeno do hiperdiagnóstico do transtorno do espectro autista (TEA), que ocorre quando o transtorno é identificado com frequência maior do que sua real relevância clínica, incluindo casos sem impacto funcional significativo. Embora o reconhecimento ampliado do transtorno tenha possibilitado inclusão e apoio a muitas pessoas, o aumento acelerado dos diagnósticos suscita questionamentos sobre critérios técnicos, potenciais usos do diagnóstico para obtenção de benefícios e a influência de fatores sociais e institucionais. Estudos epidemiológicos recentes indicam crescimento gradual da prevalência global do TEA, com dados nacionais apontando cerca de 1,2% da população diagnosticada, evidenciando maior reconhecimento e acesso ao diagnóstico. A evolução do conceito desse transtorno ampliou os critérios diagnósticos, incluindo manifestações mais leves e heterogêneas da condição, o que contribuiu para o aumento expressivo das taxas de diagnóstico ao redor do mundo. Fatores como maior conscientização social, busca por laudos escolares e pressão institucional para adequação comportamental também influenciam o aumento dos diagnósticos. O hiperdiagnóstico traz consequências negativas, como estigmatização precoce, tratamentos inadequados, impacto emocional nas famílias e distorção dos dados epidemiológicos, o que prejudica políticas públicas. Destaca-se a diferença entre diagnóstico precoce, desejável e criterioso, e diagnóstico precipitado, baseado em avaliações superficiais. Para promover um diagnóstico mais responsável, o artigo propõe a capacitação contínua de profissionais, avaliações interdisciplinares, acompanhamento longitudinal e valorização das singularidades. Além disso, defende a ampliação de políticas públicas que não dependam exclusivamente de laudos médicos para garantir apoio, priorizando as reais necessidades do indivíduo.

## Abstract (en)

This study discusses the hyperdiagnosis of autism spectrum disorder (ASD), which occurs when the identification of this disorder exceeds actual clinical relevance, including cases without significant functional impacts. Although the greater recognition of the disorder has included and supported many people, the accelerated increase in diagnoses raises questions about technical criteria, the possible use of diagnoses to obtain benefits, and the influence of social and institutional factors. Recent epidemiological studies have indicated a gradual increase in the global prevalence of ASD. Brazilian data show that about 1.2% of its population has received such a diagnosis, which evinces greater recognition and access to diagnosis. The evolution of the concept of this disorder has broadened diagnostic criteria, including milder and more heterogeneous manifestations of the condition, which has contributed to the significant increase in diagnosis rates worldwide. Factors such as greater social awareness, search for school reports, and institutional pressure for behavioral adequacy also influence increases in diagnosis. Hyperdiagnosis has negative consequences, such as early stigmatization, inadequate treatments, emotional impact on families, and distortion of epidemiological data, harming public policies. The difference between early, desirable, and judicious diagnoses and hasty ones (based on superficial evaluations) is highlighted. To promote more responsible diagnoses, this study proposes the continuous training of professionals, interdisciplinary assessments, longitudinal follow-up, and the appreciation of singularities. It also defends the expansion of public policies with no exclusive dependence on medical reports to guarantee support, prioritizing individuals’ real needs.

## Resumen

Este artículo aborda el fenómeno del hiperdiagnóstico del trastorno del espectro autista (TEA), que se produce cuando se identifica el trastorno con mayor frecuencia que su relevancia clínica real, incluidos los casos sin impacto funcional significativo. Aunque el reconocimiento ampliado del trastorno ha permitido la inclusión y el apoyo a muchas personas, el acelerado aumento de los diagnósticos plantea preguntas sobre los criterios técnicos, los usos potenciales del diagnóstico para obtener beneficios y la influencia de los factores sociales e institucionales. Estudios epidemiológicos recientes indican un aumento gradual en la prevalencia global del TEA, con datos nacionales que indican alrededor del 1,2% de la población diagnosticada, lo cual demuestra un mayor reconocimiento y acceso al diagnóstico. La evolución del concepto de este trastorno ha ampliado los criterios diagnósticos, incluidas manifestaciones más leves y heterogéneas de la enfermedad, lo cual contribuye a un significativo aumento de la tasa mundial de diagnóstico. Los factores como mayor conciencia social, la búsqueda de informes escolares y la presión institucional para la adecuación conductual también influyen en el aumento de los diagnósticos. El hiperdiagnóstico tiene consecuencias negativas, como estigmatización temprana, tratamientos inadecuados, impacto emocional en las familias y distorsión de los datos epidemiológicos, lo cual afecta las políticas públicas. Destaca la diferencia entre un diagnóstico precoz, deseable y criterioso, y un diagnóstico apresurado, basado en evaluaciones superficiales. Para promover un diagnóstico más responsable, este artículo propone la formación continua de profesionales, evaluaciones interdisciplinarias, seguimiento longitudinal y valoración de singularidades. Además, aboga por la expansión de políticas públicas que no dependan exclusivamente de los informes médicos para asegurar el apoyo, priorizando las necesidades reales del individuo.

## Introdução

O conceito de hiperdiagnóstico é empregado em contextos médicos e científicos para designar a situação em que uma condição é identificada com uma frequência maior do que sua real relevância clínica. Esse fenômeno envolve o reconhecimento de casos que provavelmente não provocariam sintomas ou impacto funcional significativo ao longo da vida do paciente ^1^ ^,^ ^2^. Na saúde pública e na epidemiologia, o hiperdiagnóstico é tema recorrente devido às consequências negativas associadas, como a realização de tratamentos desnecessários, o aumento de despesas e os possíveis prejuízos à qualidade de vida dos indivíduos afetados ^3^ ^,^ ^4^.

Nas últimas décadas, o transtorno do espectro autista (TEA) passou a ocupar um espaço central nos debates sobre saúde mental, neurodiversidade e inclusão. Esse avanço tem permitido que milhares de pessoas antes invisibilizadas sejam finalmente compreendidas em sua forma singular de estar no mundo, recebam apoio adequado e conquistem maior autonomia ^5^.

A noção de neurodiversidade constitui um marco conceitual fundamental para a compreensão contemporânea das variações no desenvolvimento neurológico. Proposta inicialmente nos campos da sociologia e dos movimentos de direitos das pessoas autistas, essa perspectiva reconhece que diferenças cognitivas, comportamentais e sensoriais, como aquelas observadas no TEA, fazem parte da diversidade humana, não sendo, por si mesmas, patológicas. Ao deslocar o foco de um modelo exclusivamente biomédico para uma abordagem que integra dimensões sociais, culturais e políticas, a neurodiversidade destaca a importância do respeito às singularidades, da redução de barreiras institucionais e da promoção de ambientes inclusivos ^6^ ^,^ ^7^.

No entanto, paralelamente a esse movimento necessário e justo, observa-se um fenômeno delicado e que merece atenção: o crescimento acelerado dos diagnósticos de TEA, muitas vezes sem o devido rigor técnico ou sem prejuízo funcional significativo ^8^ ^,^ ^9^. Esse aumento propõe a hipótese de que estar no espectro hoje pode também representar, em alguns contextos, uma espécie de passaporte para benefícios, adaptações, ou até uma identidade social que oferece sentido, acolhimento e pertencimento ^10^.

Além dos fatores clínicos e pedagógicos já mencionados, deve-se considerar também as estruturas políticas e econômicas que moldam a curva de diagnósticos ^11^. Estudos apontam que mandatos de cobertura em saúde, políticas de educação especial e distribuição desigual de recursos influenciam quem é diagnosticado e quando ^12^ ^,^ ^13^.

Por exemplo, nos Estados Unidos, com maior generosidade de seguro para o TEA, observou-se maior prevalência de diagnósticos. Ademais, a relação inicial entre diagnóstico de TEA e *status* socioeconômico mais elevado em classes altas tem se alterado, refletindo novas dinâmicas de acesso e desigualdade. Portanto, a expansão diagnóstica não pode ser entendida apenas como avanço clínico, mas como fenômeno situado em interseção entre clínica, política, economia e cultura ^9^.

Entre os benefícios desta situação, destacam-se acessos às políticas socioassistenciais, como o Benefício de Prestação Continuada, e ser considerado prioridade em serviços de saúde. Esses mecanismos, embora fundamentais para garantir direitos, também podem, em certos casos, incentivar a busca precoce e intensa por diagnósticos formais, especialmente em cenários marcados por disputas por recursos escassos ^14^.

O âmbito educacional contempla um conjunto de garantias específicas destinadas a promover a inclusão e o desenvolvimento de estudantes com TEA. Entre essas medidas, destaca-se o atendimento educacional especializado (AEE), previsto na Lei de Diretrizes e Bases da Educação Nacional (*Lei nº 9.394/1996* ^15^), o qual visa complementar e/ou suplementar o processo de escolarização por meio de serviços, recursos e estratégias pedagógicas voltadas às necessidades individuais do aluno. Além do AEE, outras políticas educacionais incluem adaptações curriculares, flexibilização de avaliações, oferta de tecnologias assistivas e a possibilidade de apoio de profissionais de apoio escolar ^15^ ^,^ ^16^ ^,^ ^17^.

Um estudo de metanálise com 66 pesquisas com amostra de mais de 21 milhões de crianças entre 2008 e 2024 apontou um aumento consolidado na prevalência global do TEA e estimou a prevalência média de cerca de 0,77% (ou 77 em cada 10 mil) em crianças de 3 a 18 anos ^18^. Complementando, o estudo *Global Burden of Disease* [Carga Global de Doenças] de 2021 relatou uma prevalência ajustada por idade de 788 por 100 mil (≈0,79%) em 2021, frente a 773 por 100 mil em 1990, revelando crescimento lento, porém contínuo ao longo de três décadas ^9^.

Além disso, a Organização Mundial da Saúde (OMS) indica uma prevalência média de aproximadamente 1% entre crianças no período de 2012 a 2021, com tendência de aumento em relação às estimativas anteriores ^19^. No Brasil, o primeiro levantamento nacional com dados autorreferidos sobre diagnóstico de autismo foi realizado pelo *Censo Demográfico* de 2022 ^20^, que incluiu pela primeira vez a pergunta: “Já foi diagnosticado(a) com autismo por algum profissional de saúde?”. Os resultados indicaram que cerca de 2,4 milhões de pessoas relataram já ter recebido esse diagnóstico, o que corresponde a 1,2% da população. Entre crianças de 5 a 9 anos, o percentual foi de 2,6%, com maiores taxas entre meninos (3,8%) do que entre meninas (1,3%).

Embora esses dados não configurem uma estimativa oficial de prevalência, oferecem um panorama relevante sobre o reconhecimento e a visibilidade do TEA no país. Para efeitos de comparação, estimativas anteriores baseadas em dados do Centro de Controle e Prevenção de Doenças dos Estados Unidos sugeriam entre 2 e 6 milhões de pessoas com TEA no Brasil até 2023, o que demonstra tanto o aumento no reconhecimento clínico, quanto a melhoria no acesso ao diagnóstico ^21^. Não se trata de negar a legitimidade do diagnóstico, nem de desacreditar aqueles que verdadeiramente enfrentam os desafios do transtorno. Tampouco se propõe aqui um discurso de suspeição generalizada ou de julgamento.

Ao contrário: a intenção é promover uma reflexão ética e crítica sobre como o autismo tem sido compreendido, diagnosticado e, em alguns casos, instrumentalizado. A valorização da neurodiversidade combate o estigma, mas pode gerar novos rótulos e transformar diagnósticos sem análise clínica aprofundada em explicações simplistas para dificuldades pessoais.

Além disso, é preciso considerar que, em determinados contextos educacionais, familiares ou institucionais, o laudo de TEA pode funcionar como requisito para acesso a apoios pedagógicos, prioridade em filas, isenções ou benefícios legais. Tal configuração não invalida essas políticas; ao contrário, trata-se de dispositivos legitimados por lutas históricas em defesa da inclusão e da equidade. Contudo, esse cenário demanda cautela para que o diagnóstico não seja banalizado ou empregado como via simplificada diante de outras questões igualmente relevantes, como dificuldades emocionais, problemas de socialização ou estilos de aprendizagem divergentes.

Portanto, o que se propõe neste estudo não é um julgamento moral, mas um convite à análise cuidadosa de um fenômeno contemporâneo: o possível hiperdiagnóstico do autismo em um contexto em que o rótulo pode representar tanto proteção quanto identidade. Como preservar a integridade clínica do diagnóstico e, ao mesmo tempo, garantir os direitos de quem realmente precisa? Essa é a questão central que precisa ser debatida com sensibilidade, responsabilidade e escuta ativa. Diante disso, este ensaio teórico tem o objetivo de refletir sobre o crescimento dos diagnósticos de autismo, questionando com sensibilidade os limites entre inclusão, reconhecimento e possíveis excessos diagnósticos na sociedade contemporânea.

## Metodologia

Este ensaio teórico-reflexivo foi desenvolvido a partir da análise crítica da literatura científica nacional e internacional, de documentos institucionais e de debates contemporâneos sobre o diagnóstico do TEA e o fenômeno do hiperdiagnóstico. Foram mobilizados textos acadêmicos relevantes, incluindo artigos originais, revisões sistemáticas, diretrizes clínicas, relatórios epidemiológicos e documentos institucionais, como a Classificação Internacional de Doenças - 11ª revisão (CID-11) e o Manual Diagnóstico e Estatístico de Transtornos Mentais (DSM-5) ^19^ ^,^ ^22^.

A proposta do ensaio é articular contribuições interdisciplinares, promovendo uma reflexão fundamentada sobre os aspectos clínicos, sociais, éticos e políticos que atravessam o aumento dos diagnósticos de TEA. A análise adotou a abordagem qualitativa, centrada na reflexão crítica dos principais conceitos, avanços e controvérsias associadas ao aumento dos diagnósticos de TEA, considerando os aspectos clínicos, sociais, éticos e políticos envolvidos.

A escolha pelo ensaio teórico reflexivo permitiu articular as evidências científicas com a análise crítica das implicações práticas e identitárias decorrentes do diagnóstico ampliado, promovendo uma discussão fundamentada e sensível acerca dos limites entre reconhecimento legítimo e possíveis excessos diagnósticos.

Este artigo foi integralmente elaborado pelos autores, sem a utilização de ferramentas de inteligência artificial generativa em nenhuma de suas etapas, incluindo redação, revisão ou formatação.

## Breve histórico e evolução do diagnóstico do TEA

O conceito de autismo foi primeiramente descrito por Leo Kanner, em 1943, como um distúrbio raro e grave do desenvolvimento ^23^. Durante grande parte do século XX, o autismo foi subdividido em categorias clínicas distintas, como autismo infantil, síndrome de Asperger e transtorno desintegrativo da infância. Foi apenas com a publicação do DSM-5, em 2013, que a noção de “espectro” foi oficialmente incorporada ao diagnóstico, unificando esses subtipos sob o termo “transtorno do espectro autista” ^22^. Essa mudança visou refletir a ampla variedade de manifestações e níveis de funcionalidade presentes na condição.

Paralelamente, a CID-11, publicada pela OMS, define o TEA como uma condição caracterizada por déficits persistentes na capacidade de iniciar e manter interações sociais recíprocas e na presença de padrões restritos, repetitivos e inflexíveis de comportamento, interesses ou atividades, com início na primeira infância e impacto significativo no funcionamento pessoal, social, educacional ou ocupacional. Tanto a CID-11 quanto o DSM-5 unificaram o diagnóstico de TEA, anteriormente fragmentado, reconhecendo o autismo como um espectro. Essa unificação contribuiu para a padronização diagnóstica, mas, por outro lado, pode haver favorecido o hiperdiagnóstico ^19^ ^,^ ^22^.

## Fatores que contribuem para o aumento dos diagnósticos

Diversos fatores têm contribuído para o aumento expressivo dos diagnósticos de TEA nas últimas décadas. Um dos principais é a ampliação dos critérios diagnósticos, especialmente após a publicação do DSM-5, que incorporou manifestações mais sutis e ampliou o entendimento do espectro ^22^.

Atualmente, sinais considerados leves ou inespecíficos, como dificuldades de socialização, comportamentos repetitivos pouco acentuados ou interesses restritos, podem ser incluídos dentro do diagnóstico. Essa ampliação, embora importante para a inclusão de casos antes negligenciados, também favorece sobreposições com outras condições, como transtorno de déficit de atenção e hiperatividade, transtornos de ansiedade social, distúrbios específicos de linguagem e até mesmo traços de personalidade atípicos, o que pode dificultar a acurácia diagnóstica ^24^.

Além disso, observa-se a crescente conscientização da sociedade civil, das famílias e das instituições escolares em relação aos sinais do TEA. O aumento da vigilância e da sensibilidade social por parte de pais, funcionárias/os de escolas, professores, psicólogos escolares e serviços comunitários, embora beneficie a detecção precoce, também pode ocasionar uma procura antecipada e, por vezes, precipitada por avaliações clínicas ^25^.

Em muitos contextos educacionais, tanto nas escolas públicas como nas privadas, observa-se a demanda crescente por laudos escolares e diagnósticos formais. Essa busca é motivada tanto por necessidades pedagógicas, como a obtenção de apoio de profissionais de apoio escolar, as adaptações curriculares ou as flexibilizações avaliativas, quanto por demandas de saúde e assistência, como o acesso a benefícios previdenciários, a prioridade em atendimentos e a inclusão em programas de apoio ^26^.

A sobreposição entre essas esferas pedagógica e clínica muitas vezes leva à interpretação de dificuldades escolares como sinais de condições neuropsiquiátricas, suscitando reflexões sobre os limites entre o cuidado educacional e o cuidado em saúde da criança e do adolescente. Esse fenômeno ocorre em contextos como escolas que solicitam relatórios para adaptações curriculares, famílias que percebem comportamentos atípicos e decidem buscar avaliação, ou instituições que incentivam rastreios nas crianças pequenas ^27^.

No caso de famílias de menor rendimento, que muitas vezes enfrentam acesso limitado a especialistas neurologistas ou psiquiatras em ambulatórios públicos, essa busca antecipada pode manifestar-se como encaminhamento via atenção primária, tentativa de obter laudo para matrícula especial, ou pressão para conseguir vagas em serviços privados ou semiprivados ^28^.

Essa dinâmica evidencia dois lados: por um lado, o benefício da maior atenção aos sinais; por outro, o risco de que avaliações sejam realizadas sem a observação longitudinal ou multidisciplinar adequada, ou antes de esgotadas as possibilidades de observação natural, resultando em diagnósticos realizados a partir de avaliações pontuais ou pressionadas pelas circunstâncias institucionais. Embora tais garantias sejam fundamentais para pessoas com deficiência, essa procura pode influenciar na elevação artificial das taxas de TEA, sobretudo quando associada a fragilidades no processo diagnóstico.

Outro aspecto relevante é a pressão social e institucional diante de comportamentos que fogem à norma. Em um cenário marcado pela padronização de condutas e pela baixa tolerância à diversidade comportamental, crianças com traços atípicos ou que apresentem dificuldades momentâneas de adaptação social são, muitas vezes, encaminhadas para avaliação especializada sem que haja prejuízo funcional significativo. Tal prática pode conduzir a diagnósticos equivocados, especialmente quando aliada à falta de capacitação técnica de alguns profissionais de saúde.

A ausência de uma abordagem multidisciplinar, baseada em critérios clínicos robustos e observação longitudinal, compromete a fidedignidade diagnóstica e aumenta o risco de hiperdiagnóstico, com impactos relevantes para o indivíduo e para as políticas de saúde pública. Esse fenômeno está associado a múltiplos fatores, como lacunas na formação profissional sobre desenvolvimento infantil e neurodiversidade, a crescente pressão social por classificações diagnósticas que deem sentido a comportamentos atípicos, a influência de dinâmicas de mercado e da sociedade que valorizam laudos para acesso a serviços e recursos, e a tendência à medicalização de aspectos da vida que poderiam ser abordados por meio de estratégias educativas ou sociais ^29^.

Tais elementos, combinados, contribuem para a expansão dos diagnósticos sem a devida ancoragem clínica e contextual. Contudo, é importante destacar que essa realidade não é homogênea: uma parcela significativa da população infantil e adolescente permanece sem acesso a especialistas e, por conseguinte, ao diagnóstico. Em muitos contextos, o acesso ocorre apenas por meio de iniciativas pontuais, como clínicas com atendimentos a valores simbólicos, ações voluntárias de profissionais em instituições religiosas ou cobertura de planos de saúde.

Esse contraste entre aqueles que conseguem e os que não conseguem acessar processos avaliativos constitui um elemento central no debate sobre o aumento dos diagnósticos, evidenciando desigualdades estruturais no acesso não apenas à saúde mental, mas também aos diversos serviços e especialidades envolvidos na identificação do TEA ^30^.

Dado que avaliações diagnósticas podem ocorrer em diferentes contextos como consultórios privados, ambulatórios de neurologia e pediatria, serviços especializados ou equipes interdisciplinares fora da Rede de Atenção Psicossocial, torna-se ainda mais evidente que essas desigualdades não são individuais, mas resultam da própria organização do sistema de saúde e de suas interfaces com a educação e com o setor privado. Isso impacta de forma significativa quem consegue ou não obter um diagnóstico formal, já que o acesso a serviços particulares costuma depender do poder aquisitivo das famílias ^31^ ^,^ ^32^.

## Consequências do hiperdiagnóstico

Embora o diagnóstico precoce do TEA seja amplamente reconhecido como um fator crucial para o desenvolvimento infantil e a implementação de intervenções eficazes, o fenômeno do hiperdiagnóstico suscita importantes preocupações clínicas, sociais e éticas. Entre os principais impactos está a rotulação precoce e indevida de crianças, o que pode gerar estigmatização ^20^. Uma vez diagnosticada com TEA, a criança pode ser percebida de maneira limitadora por familiares, professores e pela própria sociedade, comprometendo sua autoestima e influenciando negativamente as expectativas em relação ao seu potencial de aprendizagem, socialização e autonomia.

Outro efeito adverso relevante refere-se à instituição de intervenções terapêuticas inadequadas ou desnecessárias. Crianças equivocadamente incluídas no espectro podem ser submetidas a tratamentos intensivos, baseados em protocolos específicos para o autismo, que não se aplicam à sua realidade clínica. Essa situação, além de representar um custo financeiro e emocional para as famílias, implica também o uso ineficiente de recursos públicos e privados, que poderiam ser direcionados a crianças com diagnóstico confirmado e maior necessidade de suporte especializado ^33^.

O diagnóstico impreciso pode, ainda, gerar confusão e sofrimento emocional na família. Evidências indicam que o cuidado de crianças e adolescentes com deficiência recai majoritariamente sobre mulheres, especialmente mães e avós, que, diante de um diagnóstico recebido, tendem a reorganizar rotinas, responsabilidades domésticas, investimentos afetivos e expectativas futuras. Quando esse diagnóstico não reflete a real condição da criança, tais ajustes podem ser vivenciados como fonte de angústia, frustração ou culpa, ampliando a sobrecarga emocional e material dessas cuidadoras ^34^ ^,^ ^35^.

Ademais, o hiperdiagnóstico compromete a qualidade dos dados epidemiológicos, elevando artificialmente as taxas de prevalência do TEA. Essa distorção impacta o planejamento de políticas públicas baseadas em evidências, dificultando a alocação adequada de recursos e a definição de prioridades nos campos da saúde e da educação.

## Diferença entre diagnóstico precoce e diagnóstico precipitado

É importante destacar que diagnóstico precoce e diagnóstico precipitado não são sinônimos. O diagnóstico precoce é desejável quando feito com critério, por equipes multidisciplinares, com base em avaliações rigorosas e acompanhamento longitudinal ^36^. Já o diagnóstico precipitado ocorre quando, diante de sinais leves, a conclusão é apressada, sem considerar o desenvolvimento da criança ao longo do tempo e o contexto familiar, educacional e emocional. A distinção entre esses dois tipos de abordagem é essencial para garantir o cuidado ético e eficaz ^37^ ^,^ ^38^.

## Propostas para um diagnóstico mais responsável

Diante do contexto atual, torna-se imperativo implementar estratégias que minimizem os riscos associados ao hiperdiagnóstico do TEA e promovam maior rigor e responsabilidade no processo diagnóstico. A capacitação contínua de profissionais da saúde e da educação é fundamental para assegurar a compreensão adequada dos critérios diagnósticos e dos limites que definem o espectro. Essa formação deve enfatizar a necessidade de distinguir o TEA de outras condições com sintomas similares, bem como a importância de evitar diagnósticos prematuros baseados em manifestações isoladas ou transitórias ^39^.

A adoção de avaliações interdisciplinares, que integrem psicólogos, psiquiatras, fonoaudiólogos, neurologistas e pedagogos, contribui para a ampliação da acurácia diagnóstica e para a compreensão holística das demandas do indivíduo. Além disso, o acompanhamento longitudinal se apresenta como uma prática indispensável, sobretudo nos casos que evidenciam sinais leves ou controvérsias clínicas, possibilitando a observação do desenvolvimento da criança ao longo do tempo, antes da emissão de um diagnóstico definitivo ^40^.

Ainda, a valorização da individualidade e da singularidade de cada criança deve ser priorizada, evitando a utilização de rótulos diagnósticos como justificativa para comportamentos que podem ser passageiros ou influenciados pelo contexto. Por fim, é imprescindível fortalecer políticas públicas de inclusão que transcendam a exigência exclusiva de laudos médicos para o acesso a apoios pedagógicos e sociais. Tal abordagem permite que as intervenções sejam orientadas pelas necessidades reais da criança, promovendo maior equidade e eficácia no atendimento.

## Considerações finais

O aumento dos diagnósticos de TEA na atualidade é um fenômeno complexo, que deve ser analisado à luz de fatores clínicos, sociais, educacionais e culturais. Embora a identificação precoce seja um passo fundamental para a promoção do desenvolvimento e da inclusão, é preciso cautela para que o diagnóstico não se transforme em um fim em si mesmo, ou em uma resposta simplista para a diversidade comportamental humana. O desafio está em encontrar o equilíbrio entre o reconhecimento legítimo da neurodiversidade e a prevenção do excesso de medicalização, preservando a singularidade de cada indivíduo e os princípios éticos que regem a prática clínica.

## Data Availability

Não se aplica.
